# Usefulness of multiphasic MRI in assessing suitability for SIRT in treatment of liver malignancies

**DOI:** 10.1007/s00261-025-04875-2

**Published:** 2025-03-17

**Authors:** Cagri Erdim, Elife Akgun, Tevfik Guzelbey, Gulsah Yilmaz, Mehmet Hamza Turkcanoglu, Ali Dablan, Burcu Esen Akkas, Ozgur Kilickesmez

**Affiliations:** 1 University of Health Sciences Basaksehir Cam and Sakura City Hospital, Department of Radiology, Istanbul, Turkey; 2 University of Health Sciences Basaksehir Cam and Sakura City Hospital, Department of Nuclear Medicine, Istanbul, Turkey; 3 University of Health Sciences Gulhane Training and Research Hospital, Department of Radiology, Ankara, Turkey

**Keywords:** Liver neoplasms, Selective internal radiation therapy (SIRT), Magnetic resonance imaging, Single-Photon emission computed tomography

## Abstract

**Aim:**

To evaluate the predictive value of multiphasic magnetic resonance imaging (MRI) in identifying liver tumor perfusion characteristics and to compare it with hepatic artery perfusion scintigraphy findings in patients considered for selective internal radiation therapy (SIRT) with yttrium-90 (Y-90).

**Methods:**

This study included 93 patients diagnosed with primary or secondary liver cancer between May 2021 and February 2024, comprising 47 patients (27 M/20F) deemed unsuitable for SIRT and 46 patients (26 M/20F) eligible for SIRT. The relationship between multiphasic MRI and scintigraphy findings in determining perfusion of tumors was analyzed. Predictive performance was evaluated with receiver operating characteristic (ROC) analysis, and the optimal cut-off values were determined using the Youden index.

**Results:**

The SIRT unsuitable group had a lower frequency of intratumoral arterial phase hyperenhancement(APHE) (40.43% vs. 69.57%, *p* = 0.042), presence of hyperintensity on T2 sequence (72.34% vs. 95.65%, *p* = 0.026), lower lesion intensity in the portal phase (*p* = 0.033), and a lower lesion-to-liver intensity ratio in the portal phase (≤ 0.97, *p* = 0.011). The absence of intratumoral APHE [*p* = 0.049, AUC (95% CI) = 0.646 (0.508–0.783)] and a lesion-to-liver intensity ratio in the portal phase with a cut-off value of ≤ 0.97 [*p* = 0.011, AUC (95% CI) = 0.689 (0.564–0.815)] were significant predictors of SIRT unsuitability.

**Conclusion:**

Both the absence of intratumoral APHE and a lower lesion-to-liver intensity ratio in the portal phase were significant predictors of SIRT unsuitability.

**Graphical Abstract:**

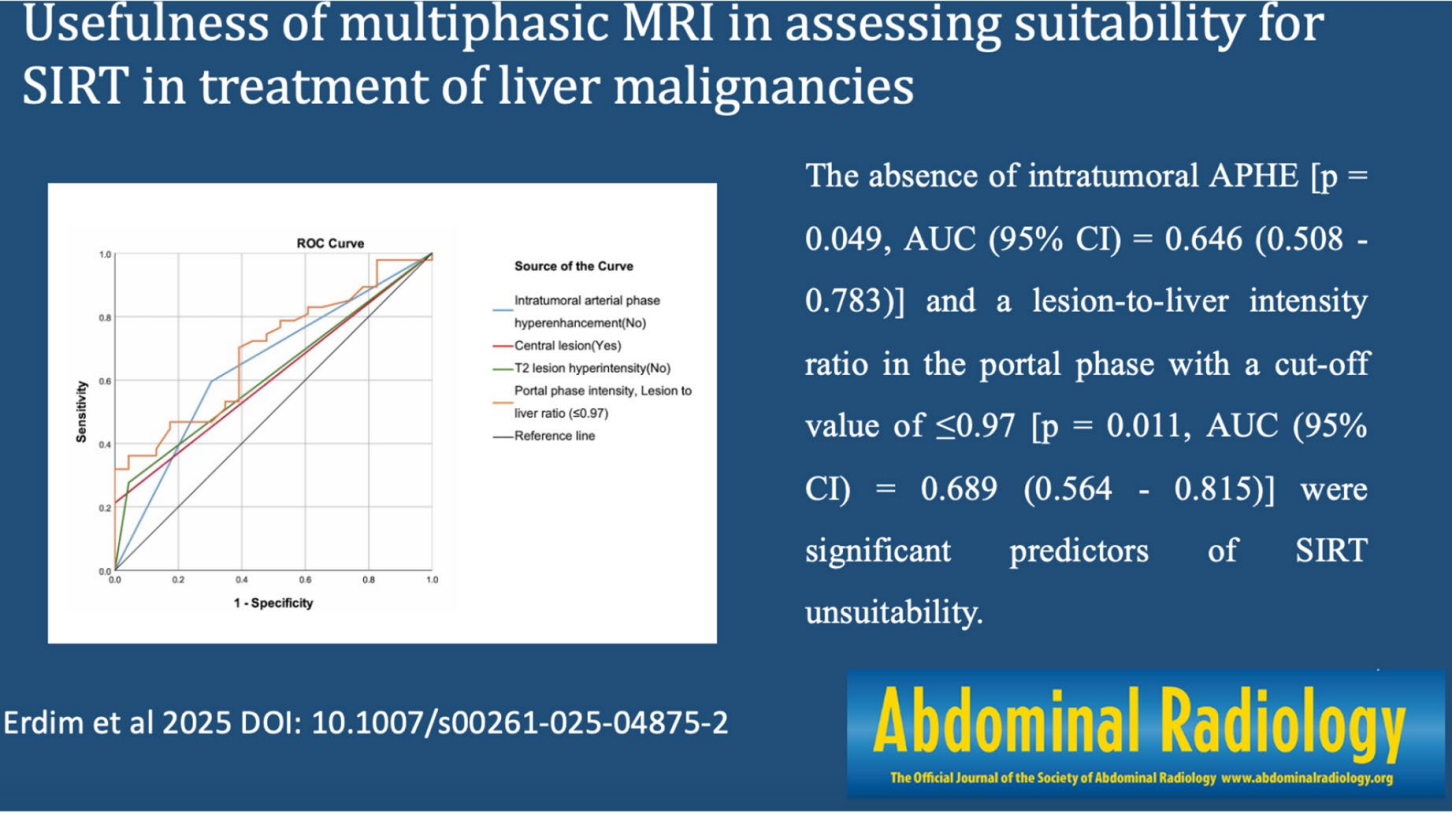

## Introduction

Primary liver cancers have a high mortality rate, ranking third among cancer-related deaths [1]. For patients who are suitable candidates for surgery, treatment options include liver transplantation or surgical resection. For the remaining patients, locoregional therapies are available.

Local-ablative options for primary and secondary liver cancers include radiofrequency ablation (RFA), microwave ablation (MWA), transarterial chemoembolization (TACE), and selective internal radiation therapy (SIRT) [2, 3]. While most of these methods are more effective in the early stages of the disease, SIRT with yttrium-90 (Y-90) loaded microspheres (glass or resin) remains a notable treatment option for advanced disease [4]. Recent studies have demonstrated that SIRT is beneficial in advanced HCC, intrahepatic cholangiocarcinoma, and colorectal cancer metastases [5–8].

Digital Subtraction Angiography (DSA) facilitates the SIRT procedure by identifying anatomical structures and variations prior to treatment [9]. After identifying the vascular structures supplying the tumor via angiography, Tc-99 m macroaggregated albumin (MAA) is selectively administered. Then planar and single-photon emission computed tomography (SPECT) is used to assess suitability for SIRT and plan the treatment [9]. SIRT cannot proceed if significant lung shunting is present, extrahepatic leakage is detected (before or during the procedure), the tumor is not sufficiently perfused, or there is intense uptake in the surrounding parenchyma [9, 10]. In this context, Tc-99 m MAA SPECT is an invasive procedure used to evaluate SIRT eligibility. It also has disadvantages such as technical challenges, high cost, limited accessibility, potential for false negatives or positives, and radiation exposure. Therefore, exploring alternative imaging methods is necessary. For instance, it is valuable to assess whether SIRT suitability can be determined by multiphasic magnetic resonance imaging (MRI), which is often routinely performed for diagnose and staging of liver cancers.

We hypothesize that certain MRI parameters can be used in predicting tumoral perfusion. For this purpose, we investigate the correlation between MRI features and Tc-99 m MAA SPECT scintigraphy findings.

## Materials and methods

### Study design and ethics

Patients with liver cancer who were testing suitability for SIRT with Y-90 were included in this retrospective study. Using Tc-99 m MAA SPECT images, patients were categorized as not eligible for the treatment due to low tumoral perfusion (Case Group) and eligible for SIRT (Control Group).Ethical approval for this study was obtained from the local ethics committee (ethics approval number: 2024-80). Written informed consent was waived by the local ethics committee in view of the retrospective nature of the study and all the procedures being performed were part of the routine care. The procedures used in this study adhere to the tenets of the Declaration of Helsinki and its later amendments.

### Study population

Patients diagnosed with primary or secondary liver cancer at our hospital between May 2021 and February 2024, who were considered for SIRT treatment, were included in this study. All patients undergoing SIRT received approval from the hepatobiliary multidisciplinary tumor board (MTB). Diagnoses and SIRT treatment indications were assessed according to current European guidelines [4]. Eligibility for SIRT treatment was assessed using Tc-99 m labeled MAA infusion via hepatic artery angiography followed by SPECT imaging. Patients with significant lung shunt on scintigraphy images, in cases where embolization of extrahepatic shunts could not be performed (identified before or during the procedure) or intense uptake in the surrounding parenchyma were deemed unsuitable for SIRT. Patients whose tumoral perfusion level compared with perfused normal liver parenchyma was low and reaching to tumoricidal radiation absorbed doses was not possible were categorized as Case Group. The remaining patients, who were deemed suitable for SIRT and underwent the procedure, were categorized as the SIRT performed Control Group.

### Outcomes

The primary outcome of the study was the ineligibility for SIRT treatment (Low tumor perfusion on Tc-99 MAA SPECT). The relationship between MRI variables and this outcome was analyzed.

#### Inclusion criteria

- Aged 18–85 years.
- Patients who were recommended for SIRT by the hepatobiliary (MTB).
- Having undergone planar and SPECT imaging following Tc-99 m-labeled MAA infusion.
- Patients in our hospital who underwent multiphasic MRI imaging prior to the procedure.

#### Exclusion criteria

- Significant lung shunt fraction (estimated lung absorbed dose over 30 Gy in single session or over 50 Gy in cumulative) detected by Tc-99 m MAA scintigraphy.
- Prior external radiotherapy to the liver.
- Patients who had previously undergone RFA, MWA, or TACE procedures.
- Gastrointestinal shunt that cannot be corrected with angiographic techniques.
- Intractable ascites.
- Elevation in liver function tests.
- Portal vein shunt and normal liver activity involvement.
- Hepatic artery spasm and dissection.
- Absence of MRI performed at our hospital prior to the procedure or the presence of significant artifacts in the MRI.
- Recent history of using immunotherapy that acts on VEGF.

### Data collection and radiological procedures

Patients demographic datas, laboratory findings, and MRI and scintigraphy images, were retrospectively obtained from the records maintained in Radiology and Nuclear Medicine departments.

### MRI protocols and related variables

Patients were instructed to fast for at least 6 h prior to the MRI examination to minimize gastrointestinal motility and reduce the risk of motion artifacts. Prior to the scan, patients were informed about the procedure and provided written consent for all procedures and data collection.

Magnetic resonance imaging was conducted on a 1.5-T MRI system (Ingenia; Philips Healthcare, Best, the Netherlands). All patients underwent liver imaging before and after the administration gadoxetic acid (Primovist; Bayer Healthcare, Berlin, Germany) at a standard dose of 0.1 mL per kilogram of body weight (0.025 mmol/mL). The contrast agent was delivered intravenously at a rate of 1 mL/s using an automatic power injector, followed by a 20-mL saline flush.

The standard MRI protocol included a T1-weighted dual-echo sequence with breath-hold, capturing both in-phase and opposed-phase images, as well as a heavily T2-weighted sequence with fat suppression, triggered by respiration. Diffusion-weighted imaging was performed using a respiratory-triggered single-shot echo-planar technique with b values set at 0, 400, and 1000 s/mm². Three-dimensional multiphasic axial volumetric images were acquired at 30 s (arterial phase), 60 s (portal phase), and 180 s (venous phase) post-contrast administration. Additional imaging was performed 20 min after contrast injection to capture the hepatobiliary phase.

### MR image analysis

All images were jointly reviewed in real-time by two radiologists with 6 and 12 years of experience in liver imaging. Discrepancies were resolved by consensus through simultaneous image review and discussion. A dedicated workstation (Intellispace Portal, Philips Healthcare, Best, the Netherlands) was used for image analysis.The following parameters were evaluated: The size of the largest lesion in contrast-enhanced series, the number of lesions, the central or peripheral location of the lesion, the presence of necrosis within the lesion, the presence of portal vein invasion, and the presence of any extrahepatic disease or vascular involvement.

For qualitative analysis, intratumoral arterial phase hyperenhancement (APHE) was visually assessed by two radiologists and classified based on its presence or absence. Similarly, T2-weighted MR images of liver lesions were evaluated qualitatively and classified according to the presence or absence of hyperintensity relative to the adjacent parenchyma.

Multiphasic contrast-enhanced, T2-weighted, and apparent diffusion coefficient (ADC) images were quantitatively evaluated. To support the results of subjective qualitative analysis, an additional quantitative analysis was performed by a diagnostic radiologist with at least 8 years of experience who did not participate in the qualitative reading session.

For the quantitative assessment of T2-weighted images, ADC sequences, and multiphasic series (including non-contrast, arterial, portal venous, venous, and hepatobiliary phases), lesion–liver signal intensity ratios were used. The contrast ratio of each lesion was calculated using the following formula: Lesion–liver contrast ratio = signal intensity of the lesion / signal intensity of the liver [11, 12]. Similarly, T2 and ADC ratios were calculated using the same approach.

For the quantitative assessment of lesion–liver ratios, lesions were identified at their largest appearance in the axial plane, and regions of interest (ROIs) of at least 300 mm² were used. The first ROIs were placed 1 mm inside the lesion borders, carefully avoiding hemorrhagic or necrotic areas to minimize susceptibility artifacts. A second ROI was placed within the tumor-free adjacent liver parenchyma in the same slice, ensuring that large blood vessels were avoided. To minimize the influence of surface coil sensitivity profiles, the ROIs within the adjacent liver parenchyma were placed at identical anatomical depths as the lesions [13]. The sizes and shapes of the ROIs were kept as consistent as possible across all measurements. Signal intensities in these ROIs were measured and recorded using the workstation.

### Tc-99 m MAA and SPECT protocols and related variables

Tc-99 m labeled MAA was prepared just before the angiography and a 185 MBq dose of Tc-99 m-MAA was slowly infused into hepatic artery branches. Perfusion scintigraphy of the hepatic artery was performed following hepatic artery angiography.

Whole-body planar and regional SPECT/CT imaging was carried out using a dual-headed gamma camera equipped with low-energy collimators (NM 860, GE HealthCare, USA). SPECT acquisition parameters were as follows: peak energy 140 keV (window: 20%), step-and-shoot protocol, 25 s/projection at a 256 × 256 matrix. The CT component was acquired by 10-mm axial sampling, 140 kpV, 2.5 mA, and at 256 × 256 matrix size. Pulmonary shunt fraction (PSF) was calculated using the following formula: PSF = Counts in the lungs / (Counts in the liver + Counts in the lungs) × 100.

Scintigraphy images were initially evaluated by two experienced nuclear medicine specialists, each managing approximately 40–50 SIRT cases per year, with only cases where both specialists reached the same conclusion being included. Extrahepatic activity uptake was excluded. Segmentations for the tumor and the liver performed using with Simplicit90Y™ Dosimetry Software (Mirada Medical LTD., Oxford, UK). The perfused normal volume was calculated by subtracting the tumor volume from the perfused volume. Volumes and predicted absorbed doses were recorded. Patients with a calculated tumor dose below 150 Gy on voxel-based dosimetry were identified to be ‘unsuitable’ for SIRT treatment.

Counts and volumes for perfused liver, perfused tumor, perfused normal liver, whole liver, normal liver were calculated. Counts were corrected using with volumes (Counts/volumes) Tumor-to-normal parenchyma perfusion ratio, and tumor-to-total parenchyma perfusion ratio were examined.

### Statistical analysis

All analyses were performed on SPSS v25 (IBM Corp., Armonk, NY, USA). Two-tailed p-values of less than 0.05 were considered statistically significant. The Shapiro-Wilk test was used to assess normality. Descriptive statistics were presented as mean ± standard deviation for normally distributed continuous variables, median (25th percentile − 75th percentile) for non-normally distributed continuous variables, and with frequency (percentage) for categorical variables. Normally distributed variables were analyzed using the Student’s t-test. Non-normally distributed variables were analyzed using the Mann-Whitney U test. Categorical variables were analyzed using chi-square tests or the Fisher’s exact test or the Fisher-Freeman-Halton test. Prediction performance was assessed using receiver operating characteristic (ROC) curve analysis (area under the curve; AUC) and the cut-off values were based on Youden J statistic.

## Results

**Fig. 1:**
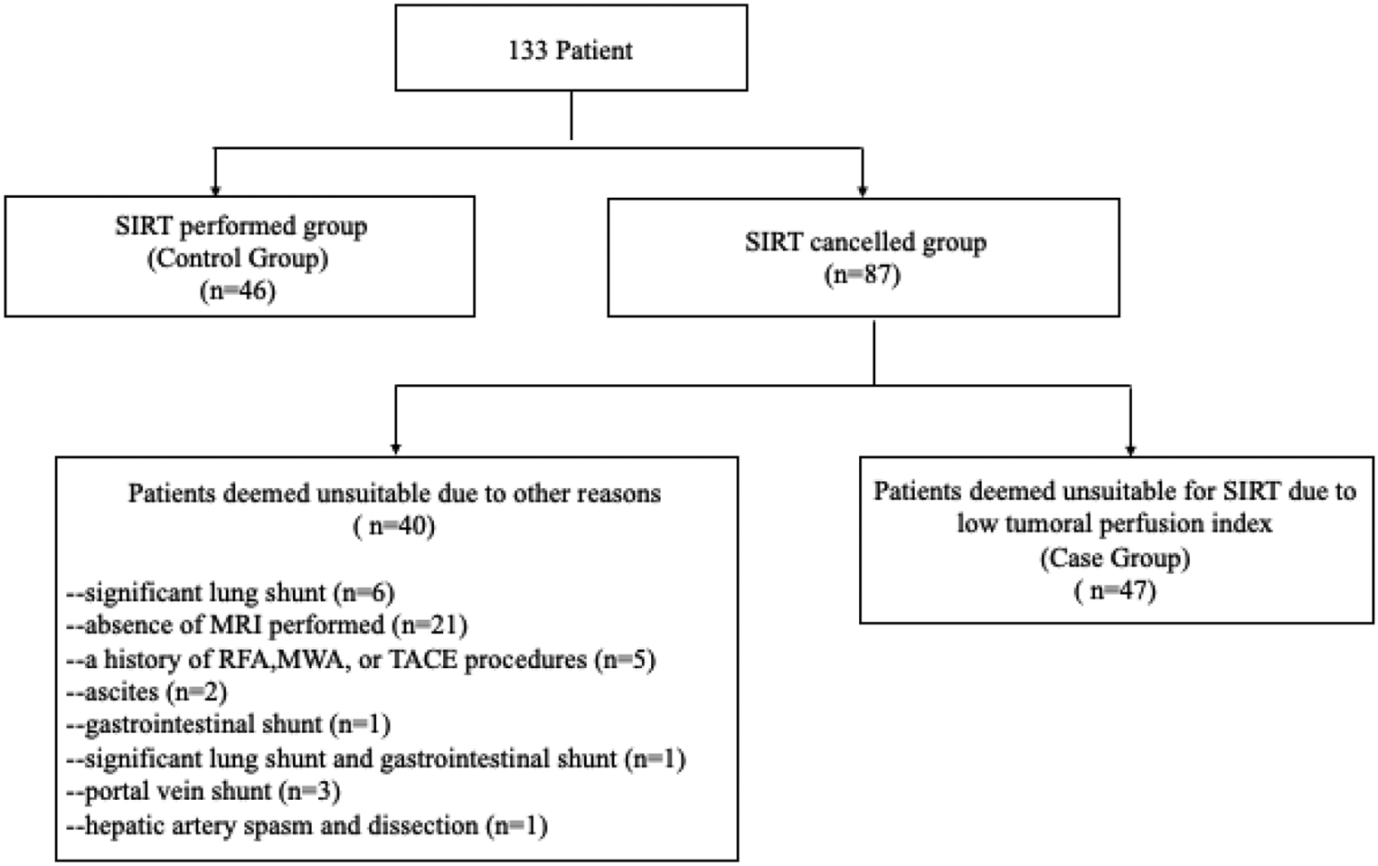
The flowchart of the study **(MAA**: Tc-99 m-macroaggregated albumin; **MRI**: Magnetic resonance imaging; **MWA**: Microwave ablation **RFA**: Radiofrequency ablation; **TACE**: Transarterial chemoembolization; **SIRT**: Selective internal radiation therapy

**Fig. 2:**
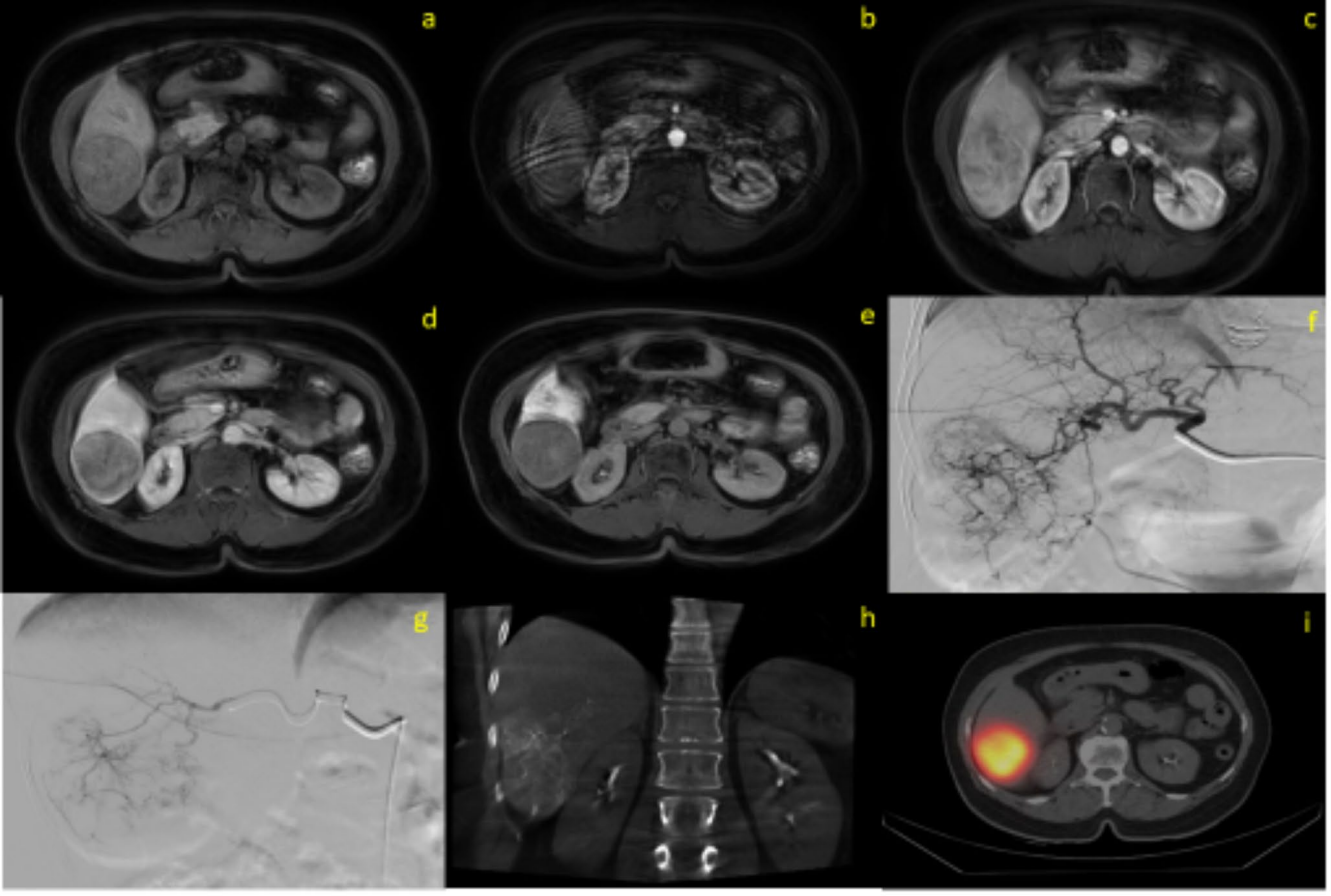
Imaging of a 63-year-old female patient with an 46 mm HCC lesion, demonstrating MAA uptake in segment 6. (**a**-**e**) Precontrast phase (**a**), Arterial phase (**b**), Portal venous phase (**c**), Venous phase (**d**) and Hepatobiliary phase (**e**) contrast-enhanced T1-weighted MRI image of the lesion in segment 6; (**f**, **g**) Angiographic images of the main hepatic artery (**f**) and the segmental artery with MAA administration (**g**); (**h**) Cone beam CT image of the segmental artery where MAA was administered; (**i**) SPECT imaging after MAA injection. **(CT**: Computed tomography; **HCC**: Hepatocellular carcinoma; **MAA**: Tc-99 m-macroaggregated albumin; **MRI**: Magnetic resonance imaging; **SPECT**: Single-photon emission computed tomography)

**Fig. 3:**
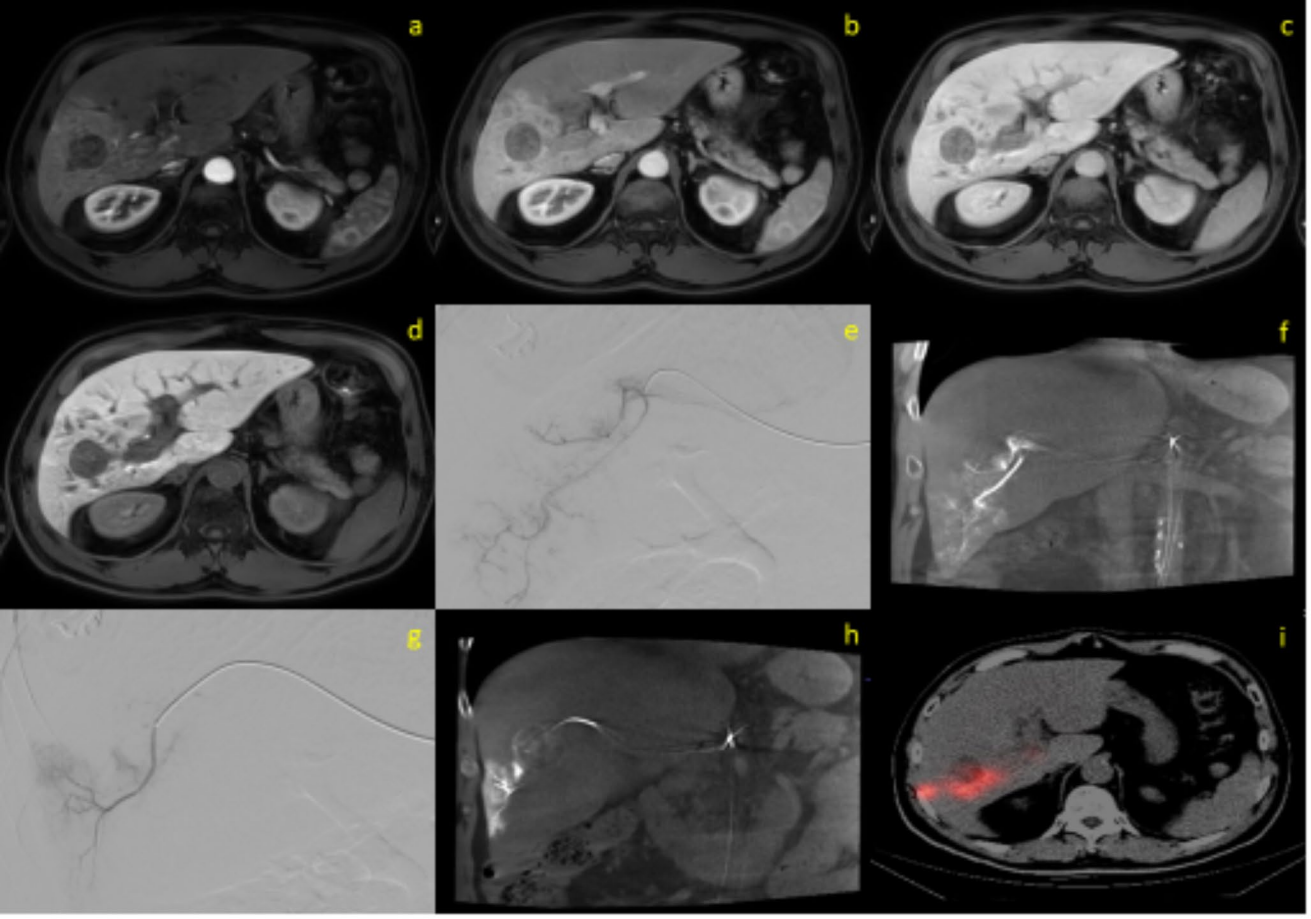
A 53-year-old patient with an 34 mm HCC lesion in segments 5–6 was deemed unsuitable for treatment following split-dose MAA injection through two separate segmental arteries. (**a**-**d**) Arterial phase (**a**), Portal venous phase (**b**), Venous phase (**c**) and Hepatobiliary phase (**d**) contrast-enhanced T1-weighted MRI image of the lesion in segment 5–6; (**e**, **f**) Angiographic (**e**) and cone beam CT (**f**) images of the segmental artery where the first MAA was administered; (**g**, **h**) The segmental artery in which the second MAA was administered, depicted in both angiographic (**g**) and cone beam CT (**h**) images; (**i**) SPECT/CT image demonstrating MAA uptake predominantly in the posterior part of the lesion and within the normal parenchyma, without complete coverage of the entire mass. **(CT**: Computed tomography; **HCC**: Hepatocellular carcinoma; **MAA**: Tc-99 m-macroaggregated albumin; **MRI**: Magnetic resonance imaging; **SPECT**: Single-photon emission computed tomography)

Forty-six patients for the control group (SIRT performed), 47 patients for the case group(SIRT canceled) were included in this retrospective study Fig. 1. The mean age of the case group (61.30 ± 9.65) and the control group (62.52 ± 15.60) was similar (*p* = 0.732). There was no significant difference in sex distribution between the groups (*p* = 1.000). The tumors were classified into two groups: primary cancers [hepatocellular carcinoma (HCC), cholangiocarcinoma] and secondary cancers (metastases). Among the patients, 49 (52.68%) had primary liver cancer, while 44 (47.32%) had secondary liver cancer. Between two groups no significant difference was detected in terms of origin of the tumors (*p* = 0.322). Size of tumors were similar of the groups (*p* = 0.896). Compared to SIRT-suitable patients, those unsuitable for SIRT had a significantly lower percentage of intratumoral APHE (*p* = 0.042), presence of T2 hyperintensity (*p* = 0.026), lesion intensity in the portal phase (*p* = 0.033), lesion-to-liver intensity ratio in the portal phase (*p* = 0.011), and tumor SPECT count per volume-to-normal parenchyma perfusion SPECT count per volume (*p* < 0.001). Patients unsuitable for SIRT also had significantly higher frequency of centrally located lesions (*p* = 0.025). Patients all demographic and clinic datas were showen in Table 1. Examples patients were shown in Figs. 2 and 3.

**Table 1:** Summary of demographics, MRI and SPECT findings with regard to groups

	SIRT		
	Cancelled Case Group (*n* = 47)	Performed Control Group (*n* = 46)	p
Age	61.30 ± 9.65	62.52 ± 15.60	0.732^†^
Sex			
Male	27 (57.45%)	26 (56.52%)	1.000^#^
Female	20 (42.55%)	20 (43.48%)	
Malignancy			
Primary malignancy	23 (48.94%)	26 (56.52%)	0.732^#^
Secondary malignancy	24 (51.06%)	20 (43.48%)	
Malignancy subtype			
Hepatocellular carcinoma	21 (44.68%)	18 (39.13%)	0.322^¶^
Cholangiocarcinoma	2 (4.26%)	8 (17.39%)	
Sarcoma cancer	2 (4.26%)	0 (0.00%)	
Colon carcinoma	10 (21.28%)	14 (30.43%)	
Rectum cancer	4 (8.51%)	0 (0.00%)	
Breast cancer	2 (4.26%)	0 (0.00%)	
Bladder cancer	0 (0.00%)	2 (4.35%)	
Neuroendocrine cancer	3 (6.38%)	4 (8.70%)	
Pancreas cancer	2 (4.26%)	0 (0.00%)	
Cervical cancer	1 (2.13%)	0 (0.00%)	
Number of lesions			
One	14 (29.79%)	18 (39.13%)	0.609^#^
Two or more	33 (70.21%)	28 (60.87%)	
Largest lesion size (mm)	49.5 (36–83)	46.5 (30–93)	0.896^‡^
Intratumoral APHE	19 (40.43%)	32 (69.57%)	**0.042** ^**#**^
Central lesion	10 (21.28%)	0 (0.00%)	**0.025** ^**§**^
Necrosis	12 (25.53%)	8 (17.39%)	0.646^#^
Portal vein invasion	5 (10.64%)	12 (26.09%)	0.159^§^
Presence of T2 hyperintensity	34 (72.34%)	44 (95.65%)	**0.026** ^**§**^
ADC			
Lesion	1.06 (0.89–1.36)	1.09 (0.94–1.17)	0.804^‡^
Liver	1.22 (1.12–1.38)	1.15 (0.98–1.28)	0.150^‡^
Lesion to liver ratio	0.85 (0.69–1.16)	0.95 (0.80–1.08)	0.545^‡^
T2 intensity			
Lesion	895 (739–1185)	857 (761–1125)	0.990^‡^
Liver	527 (444–647)	541 (494–678)	0.368^‡^
Lesion to liver ratio	1.64 (1.35–2.09)	1.51 (1.29–1.88)	0.557^‡^
Precontrast intensity			
Lesion	905.98 ± 216.22	865.70 ± 170.44	0.437^†^
Liver	1157.87 ± 233.40	1176.78 ± 253.71	0.758^†^
Lesion to liver ratio	0.75 (0.69–0.82)	0.77 (0.66–0.82)	0.689^‡^
Arterial intensity			
Lesion	1139.26 ± 322.43	1141.39 ± 349.20	0.980^†^
Liver	1254.30 ± 283.13	1218.96 ± 233.55	0.606^†^
Lesion to liver ratio	0.87 (0.74–1.05)	0.89 (0.77–0.99)	0.856^‡^
Portal intensity			
Lesion	1354 (1018–1687)	1643 (1189–1923)	**0.033** ^**‡**^
Liver	1691 (1375–1920)	1643 (1413–1944)	0.915^‡^
Lesion to liver ratio	0.81 (0.65–1.02)	1.02 (0.79–1.11)	**0.011** ^**‡**^
Venous intensity			
Lesion	1483.53 ± 507.76	1633.09 ± 588.21	0.276^†^
Liver	1769 (1494–2162)	1890 (1490–2375)	0.783^‡^
Lesion to liver ratio	0.78 ± 0.22	0.88 ± 0.27	0.138^†^
Hepatobiliary (20th minute) intensity			
Lesion	607 (482–866)	545 (404–800)	0.243^‡^
Liver	1027.96 ± 296.92	1015.83 ± 330.31	0.879^†^
Lesion to liver ratio	0.63 ± 0.12	0.58 ± 0.14	0.098^†^
Hepatopulmonary shunt, %	2.6 (1.0–5.8)	3.6 (1.2–7.0)	0.778^‡^
Tumor count per volume (A) (cm^3^)	2663.02 (739.07–5012.68)	3691.68 (1349.33–13815.73)	0.142^‡^
Normal perfused liver count per volume (B) (cm^3^)	2216.63 (1102.53–4270.12)	1296.91 (641.89–3709.95)	0.191^‡^
Total perfused liver count per volume (C) (cm^3^)	2336.60 (1017.11–3816.59)	2656.19 (1207.49–6532.54)	0.392^‡^
Tumor to normal perfused liver ratio (A/B)	1.54 ± 1.11	2.66 ± 0.97	**< 0.001** ^**†**^
Tumor to total perfused liver ratio (A/C)	1.19 (0.79–1.71)	1.35 (1.15–1.55)	0.191^‡^

Descriptive statistics were presented using mean ± standard deviation for normally distributed continuous variables, median (25th percentile − 75th percentile) for non-normally distributed continuous variables and frequency (percentage) for categorical variables

† Student’s t test, ‡ Mann Whitney U test, # Chi-square test, § Fisher’s exact test, ¶ Fisher-Freeman-Halton test.

Abbreviations; APHE: Arterial phase hyperenhancement ADC: Apparent Diffusion Coefficient, SIRT: Selective Internal Radiation Therapy

**Fig. 4:**
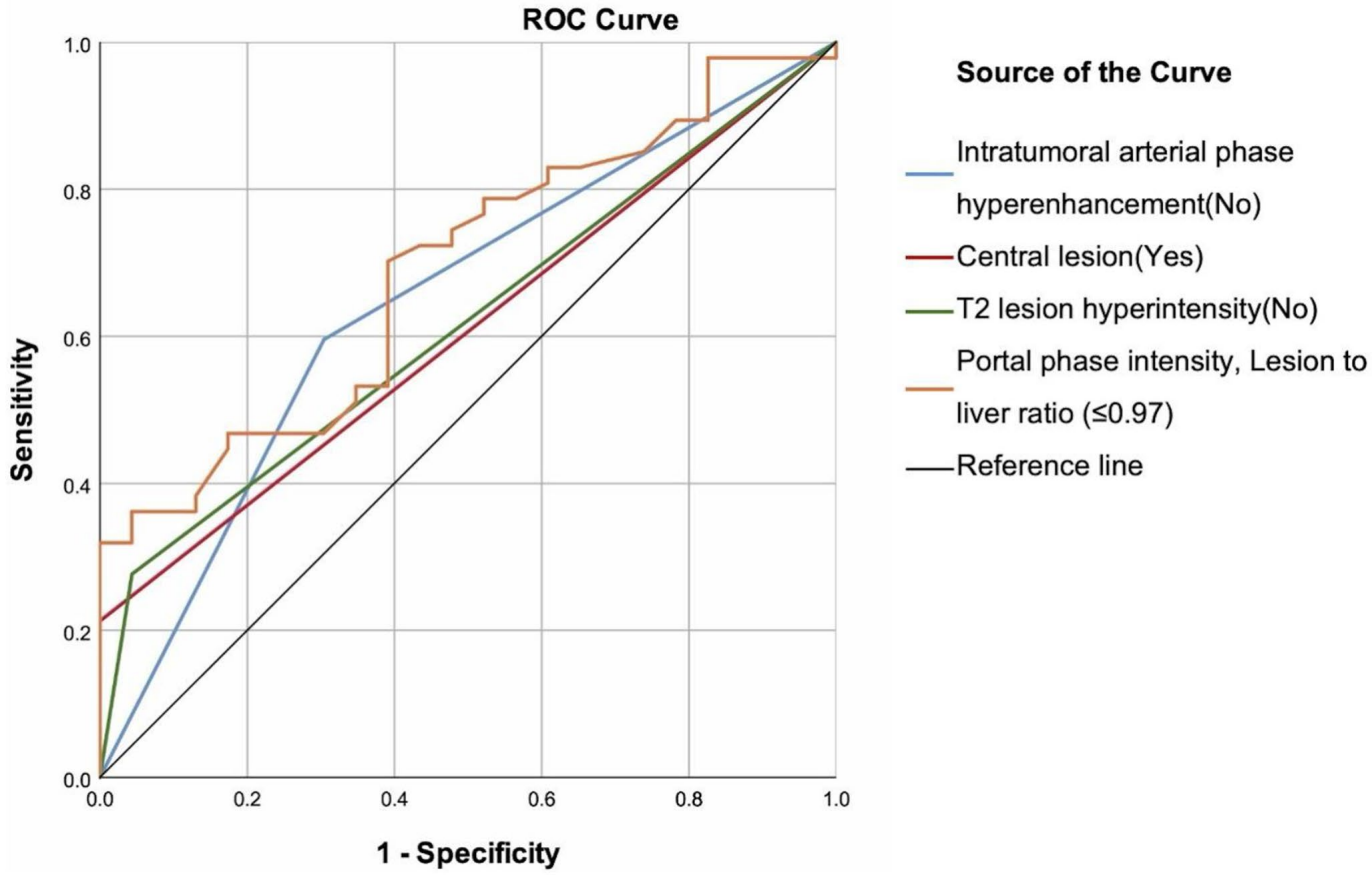
ROC curves of the magnetic resonance imaging findings to predict patients not suitable for selective internal radiation therapy

The absence of intratumoral APHE [Sensitivity = 59.57%, Specificity = 69.57%, AUC (95% CI) = 0.646 (0.508–0.783), *p* = 0.049] and the lesion intensity-to-liver intensity ratio in the portal phase [cut-off: ≤0.97; Sensitivity = 70.21%, Specificity = 60.87%, AUC (95% CI) = 0.689 (0.564–0.815), *p* = 0.011] were significant in identifying patients unsuitable for SIRT (Table 2) (Fig. 4).

**Table 2:** Performance of variables to predict patients who were not eligible for SIRT, ROC curve analysis

	Intratumoral APHE	Central lesion	T2 lesion hyperintensity	Portal intensity, Lesion to liver ratio
Cut-off	No	Yes	No	≤ 0.97
Sensitivity	59.57%	21.28%	27.66%	70.21%
Specificity	69.57%	100.00%	95.65%	60.87%
Accuracy	62.86%	47.14%	50.00%	67.14%
PPV	80.00%	100.00%	92.86%	78.57%
NPV	45.71%	38.33%	39.29%	50.00%
AUC (95% CI)	0.646 (0.508–0.783)	0.606 (0.473–0.739)	0.617 (0.484–0.749)	0.689 (0.564–0.815)
p	**0.049**	0.150	0.115	**0.011**

Abbreviations; APHE: Arterial phase hyperenhancement, AUC: Area under ROC curve, CI: Confidence interval, NPV: Negative predictive value, PPV: Positive predictive value, ROC: Receiver operating characteristic, SIRT: Selective internal radiation therapy

## Discussion

In this study, we investigated the predictive value of multiphasic MRI in assessing liver tumor perfusion characteristics and its potential role in determining suitability for selective internal radiation therapy (SIRT) with Yttrium-90 (Y-90). Our findings revealed that the absence of APHE, a low lesion-to-liver intensity ratio in the portal phase (cut-off ≤ 0.97), central tumor localization, and T2 hyperintensity were significant predictors of SIRT unsuitability. Among these, the lesion-to-liver intensity ratio in the portal phase demonstrated the highest AUC (0.689), suggesting that multiphasic MRI could serve as a valuable non-invasive tool for assessing tumor perfusion and optimizing patient selection in SIRT.

SIRT with Y-90 loaded microspheres is a well-established treatment option for patients with chemo resistant and unresectable liver cancer. These microspheres are selectively injected into the hepatic artery to deliver high radiation doses directly to the tumor [14]. However, large-scale randomized controlled trials have not shown significant improvements in survival for patients treated with resin microspheres, and one possible reason for this may be suboptimal dosing strategies [9, 15–19].

The angiography approach involves the injection of a mock particle, Tc-99 m-MAA, to simulate and visualize the expected distribution of therapeutic microspheres in SIRT. The primary reasons for employing this simulation procedure with Tc-99 m-MAA are as follows: (i) to assess the vascularity of the tumor and determine its perfusion status, (ii) to predict the potential distribution and localization of radioactive particles, thereby enhancing the effectiveness and safety of the treatment, and (iii) to determine the hepatopulmonary shunt ratio, assessing the risk of radioactive particles escaping into the lungs [9, 20, 21]. Additionally, if there are hepatointestinal shunta that cannot be successfully occluded with coil embolization and could allow Tc-99 m-MAA to escape outside the liver, SIRT may be risky and should not be performed [10].

Although Tc-99 m MAA scintigraphy is arguably the most reliable method for ensuring SIRT safety, it is not without various disadvantages. Variations in vascular anatomy or technical errors can lead to false positive or negative results. It is also important to note that scanning with Tc-99 m MAA exposes the patient to additional radiation. Incorrect assessment of the hepatopulmonary shunt ratio can lead to errors in treatment planning. The cost of Tc-99 m MAA and accessibility issues in certain regions can limit its use [9, 14, 22]. Wondergem and et al. claimed that the distribution of Tc-99 m MAA does not accurately predict the final distribution of Y-90 activity [14]. They also reported several factors that could cause discordance between the distribution of Tc-99 m MAA and Y-90 microspheres, including interval differences in catheter position, physiological differences in hepatic blood flow, differences in the size and morphology of Tc-99 m-MAA particles and Y-90 microspheres, tumor histopathology, and tumor load [14]. These drawbacks highlight the need for diagnostic tools that are cheaper, safer, easier to apply, and offer more reliable results in determining patients suitable or unsuitable for SIRT.

In studies assessing the absorbed dose threshold in radioembolization, tumor size and location have been shown to significantly impact treatment outcomes. Therefore, multiple sessions and detailed technical evaluations might be required to ensure a complete response [23–25]. In the context of HCC, gastrointestinal stromal tumors, or melanoma-derived liver metastases, targeted therapies such as antiangiogenic agents and tyrosine kinase inhibitors can paradoxically increase tumor size due to hemorrhage or necrosis [26, 27]. MRI is particularly effective in identifying intratumoral changes, including hemorrhage and necrosis [28]. In our study, we carefully delineated ROI to focus on the solid components of the tumors, avoiding areas of necrosis and hemorrhage. Tumor sizes were similar between the two groups, ensuring that the angiographic procedure was technically influenced equally, minimizing the impact of confounding factors such as intratumoral hemorrhage and necrosis on MRI measurements.

Our study demonstrated that patients unsuitable for selective internal radiation therapy (SIRT) had a significantly lower percentage of lesion presence of T2 hyperintensity (*p* = 0.026). T2 hyperintensity is generally linked to increased water content, whereas T2 hypointensity is associated with fibrosis, necrosis, and low vascularity [29]. Granata et al. reported that ICC lesions with central fibrous stroma appear hypointense on T2-weighted imaging, influencing their vascular patterns and post-contrast enhancement [30]. Additionally, tumors with higher arterial enhancement on preoperative imaging were shown to have less necrosis and fibrosis and better survival outcomes, highlighting the impact of vascularity on prognosis [31]. Furthermore, arterial perfusion plays a critical role in tumor response to radioembolization. Prior studies have indicated that arterially hypoperfused tumors respond poorly to Y-90 radioembolization due to inadequate microsphere deposition within the lesion [32]. Our results suggest that presence of T2 hyperintensity could serve as a biomarker for SIRT eligibility. The hypointense appearance of some necrotic and fibrotic tumors on T2-weighted MRI due to low water content suggests that these tumors may have reduced perfusion and may not be suitable for SIRT.

The effectiveness of selective internal radiation therapy (SIRT) in hepatic malignancies is closely tied to the arterial hypervascularization of tumors, as highlighted by Weber et al. [33]. Arterial phase hyperenhancement (APHE) serves as a well-established imaging biomarker for hypervascular tumors, reflecting the preferential arterial supply to liver tumors that enables targeted radiation delivery while sparing healthy liver tissue [34–36]. In our study, the absence of intratumoral APHE emerged as a significant predictor of SIRT unsuitability. These findings are consistent with prior research demonstrating that CT perfusion imaging-based assessment of arterial tumor perfusion can predict both short-term morphologic response and one-year SIRT. Specifically, an arterial perfusion threshold exceeding 16 mL per 100 mL/min has been identified as a critical determinant of therapy response and survival outcomes [35]. Collectively, these results highlight the necessity of meticulous arterial phase analysis in multiphasic imaging to optimize patient selection for SIRT and ensure therapeutic efficacy.

In our study, patients deemed unsuitable for SIRT had a significantly higher frequency of centrally located lesions (*p* = 0.025). Hemodynamic differences between central and peripheral zones may be a key factor affecting SIRT efficacy [37]. Previous studies have demonstrated that central and peripheral tumors exhibit distinct vascular characteristics, which may influence treatment response [38, 39]. Specifically, in patients undergoing TACE for HCC, centrally located tumors have been reported to show lower complete response rates compared to peripheral tumors [39]​.

Several mechanisms may explain the lower response rates observed in central lesions. First, the vascular anatomy in central regions is more complex, and arterial supply to these tumors may be more variable compared to peripheral tumors​. Second, centrally located tumors are more likely to be supported by collateral arteries, which can reduce the efficacy of intra-arterial therapies​. Third, heterogeneous tumor perfusion in centrally located lesions may prevent uniform distribution of embolic agents or radioactive particles, further limiting treatment effectiveness​ [39]. These findings suggest that multiphasic MRI should be interpreted with caution when assessing SIRT eligibility for centrally located tumors, and additional dose optimization or supplementary evaluation methods may be required for these patients.

This study has several limitations that should be acknowledged. First, it was conducted in a single institution with a relatively small sample size, which may limit the generalizability of the findings. Prospective studies with larger cohorts and standardized imaging protocols are needed to validate these results. Additionally, patients with different types of liver malignancies (e.g., hepatocellular carcinoma, cholangiocarcinoma, and liver metastases) were analyzed as a single cohort, despite their distinct enhancement patterns, perfusion characteristics, and biological behavior. This heterogeneity, combinedwith physiological variations in hepatic blood flow and tumor burden, may have influenced both the distribution of Tc-99 m MAA/Y-90 microspheres and MRI parameters such as arterial phase hyperenhancement (APHE), lesion-to-liver intensity ratio, and the presence of T2 signal. Since these factors were not analyzed separately, their exact impact remains unclear.

Furthermore, despite efforts to minimize their influence, regenerative nodules and background parenchymal changes associated withchronic liver disease could not always be fully excluded from ROI measurements, potentially introducing variability in MRI-derived signal intensity assessments. Future studies with larger, well-defined subgroups and advanced imaging protocols, including perfusion-based imaging techniques, may help clarify these effects and further refine the role of multiphasic MRI in assessing SIRT eligibility.

## Conclusion

In conclusion, for liver cancer patients scheduled for SIRT with Y-90, multiphasic MRI, particularly portal phase imaging, may provide valuable and non-invasive information for patient selection prior to invasive hepatic artery angiography. The ability to predict SIRT suitability using MRI could reduce the need for additional invasive procedures and lower treatment costs. However, these findings require validation through larger, prospective studies, ideally incorporating advanced imaging techniques such as perfusion MRI or radiomics, before clinical implementation. This study highlights the potential of multiphasic MRI as a promising tool for optimizing patient selection in SIRT, contributing to the development of more personalized and effective treatment strategies.

## Data Availability

No datasets were generated or analysed during the current study.
